# RNA-Seq Analysis Reveals Potential Genes Involved in Plant Growth Regulator-Induced Ovary Development in Male Kiwifruit (*Actinidia eriantha*)

**DOI:** 10.3390/plants14050703

**Published:** 2025-02-25

**Authors:** Rong Fu, Min Zhang, Feng Wei, Miaomiao Lin, Jinbao Fang, Ran Wang, Yukuo Li, Jinyong Chen, Leiming Sun, Xiujuan Qi

**Affiliations:** 1 School of Agricultural Sciences, Zhengzhou University, Zhengzhou 450001, China; furong2029@163.com (R.F.); weifeng0108@163.com (F.W.); 2 National Key Laboratory for Germplasm Innovation & Utilization of Horticultural Crops, Zhengzhou Fruit Research Institute, Chinese Academy of Agricultural Sciences, Zhengzhou 450009, China; zhangmin1862@163.com (M.Z.); linmiaomiao@caas.cn (M.L.); fangjinbao@caas.cn (J.F.); wangran@caas.cn (R.W.); liyukuo@caas.cn (Y.L.); chenjinyong@caas.cn (J.C.); 3 National Key Laboratory of Cotton Bio-Breeding and Integrated Utilization, Institute of Cotton Research of Chinese Academy of Agricultural Sciences, Anyang 455000, China; 4 Zhongyuan Research Center, Chinese Academy of Agricultural Sciences, Xinxiang 453500, China; 5 Chuxiong Yunguo Agriculture Technology Research Institute, Chuxiong 675000, China

**Keywords:** *Actinidia eriantha*, PGR, induction, ovary, RNA-Seq

## Abstract

Kiwifruit is a dioecious woody liana fruit tree, and the non-fruitfulness of male plants leads to a great deal of blindness in the selection of male plants in crossbreeding. In this study, we induced the development of male plant ovary by externally applying plant growth regulators (PGRs) and performed histological observation, phytohormone content determination and transcriptome analysis on the abortive ovary of the male kiwifruit (Con), the ovary of the female kiwifruit (Fem) and the PGR-induced developing ovary of the male kiwifruit (PT). Histological analysis showed that the Con ovary was devoid of ovules and the carpels were atrophied, the Fem ovary had ovules and the PT ovary was devoid of ovules, but the carpels developed normally and were not atrophied. Endogenous phytohormone content measurements displayed higher levels of trans-zeatin (tZT) in PT and Fem than Con, and lower levels of gibberellin (GA3) and abscisic acid (ABA) than Con. Transcriptome analysis revealed significant differences in many key genes in the cytokinin and auxin pathways, which were consistent with the results of phytohormone content measurements. Meanwhile, the genes related to carpel development, SPT (*DTZ79_04g03580*) and SK41 (*DTZ79_19g04340*), were highly expressed in PT, suggesting that they may play a key role in PGR-induced development of the ovary in male kiwifruit. These results provide information for elucidating the potential regulatory network of PGR-induced ovary development in male flowers and contribute to further identification of valuable target genes.

## 1. Introduction

Kiwifruit (*Actinidia* Lindl), one of the most successful examples of plant cultivation and domestication from wild to commercial cultivation in the 20th century, belongs to the genus *Actinidia* and is also a major fruit crop consumed worldwide [1,2]. Most *Actinidia* species are dioecious, with male and female flowers similar in morphology and structure during the early developmental stages. However, in male flowers, the pistil stops developing soon after the stigma develops, and many stamens surround a rudimentary ovary with a necrotic pistil [3]. Due to the non-development of the kiwifruit male ovary, we cannot understand the traits of male fruit, so there is great uncertainty in the selection of male plants in crossbreeding, and it is difficult to analyze the inheritance of traits and variation in the progeny while crossbreeding has gradually occupied an increasingly important position in the current kiwifruit breeding practice [4]. Therefore, it is imperative to find a method to promote the development of the male ovary in kiwifruit and study its specific mechanism.

Genome sequencing combined with transcriptome analysis was exploited; two sex-determining genes on the Y chromosome in kiwifruit, *Shy Girl* (*SyGl*) and *Friendly Boy* (*FrBy*), were isolated and identified, which solved the problem of sex determination in *A. chinensis* and responded to the well-known ‘two-mutation model’ hypothesis [5,6]. This hypothesis suggests that the evolution from an autosomal chromosome to an effective Y chromosome involved at least two factors: a dominant mutation in the suppressor of feminization (SuF), and a recessive mutation that causes male sterility [7]. *SyGl*, localized in a genome-specific region of the Y chromosome, may originate from a clade-specific gene duplication event and is a type C cytokinin response regulator. Functional transgenic analyses in *Arabidopsis thaliana* and tobacco revealed that *SyGl* is a dominant repressor of carpel development and inhibits this process by regulating the cytokinin response pathway [5]. *FrBy*, encoding a FAS1 structural protein, is a male-promoting factor that regulates pollen fertilization in female flowers by delaying apoptosis in anther petiole cells. Transgenic expression of *FrBy* produced kiwifruit hermaphrodite lines with restored androgenic function and complemented defects in pollen tube development, validating the role of *FrBy* as a malefactor in kiwifruit sex determination [6]. Recently, it has been found that the kiwifruit feminization suppressor of *SyGl* is a cytokinin-responsive regulator; moreover, kiwifruit male carpel development can be partially restored by externally applied cytokinin [8].

It is well known that phytohormones, especially cytokinins and auxin, can regulate many plant growths and developmental processes [9,10,11], and they usually dynamically co-regulate biological processes in a complementary mode [12]. Cytokinins are key initiators of cell division and differentiation, and the most common endogenous cytokinin in higher plants is trans-zeatin (tZT), which plays an important role in gynoecium development and in the formation of the carpel marginal meristem (CMM) [13]. In *Arabidopsis thaliana*, for example, the CMM is located on the proximal axial side of the carpel margin, which gives rise to tissues and organs within the pistil, including the placenta, ovules, septum and transmitting tract [14]. *INDEHISCENT* (*IND*), *SHATTERPROOF1* (*SHP1*) and *SHP2* are key regulators in the development of CMM. *Ind* and *shp1 shp2* are mutants in which the flap margins are cleaved, and local application of cytokinins partially restored CMM function, demonstrating that cytokinins mediate the effects of *IND* and *SHP1/2* on CMM development and function [15]. Cytokinins are also involved in female gametophyte development, and cytokinin receptors in sporophyte tissues are essential for female gametophyte development in *Arabidopsis* [16,17]. In addition, auxin plays an important role in the apical–basal pattern of the gynoecium [18]. Auxin is synthesized locally at the apex of the gynoecium and then transported toward the base, resulting in high levels of auxin in the apical portion and a gradient of auxin concentration from the apex to the base [19]. High levels of auxin promoted the development of the style at the apex, medium levels of auxin promoted the development of the ovary, and low levels of auxin promoted the development of the gynophore at the base [18]. The apical–basal pattern of *Arabidopsis* leads to severe defects due to disruption of polar auxin transport, whereas externally applied cytokinins can influence the polar auxin transport of the developing gynoecium to act in the apical–basal pattern [20]. These results suggest that cytokinins and growth hormones work together to influence the development of the gynoecium [21].

In this study, we used cytokinin and auxin to formulate a plant growth regulator (PGR) treatment solution, and induced the development of *A. eriantha* male ovary by exogenous use of the treatment solution. The changes in histology, phytohormone and transcriptome levels of male ovaries before and after the treatment and female ovaries were investigated, and the potential genes involved in the PGR-induced development of kiwifruit male flower ovary were explored. The results of this study provide a reference for the understanding of hormone-induced ovary development in male flowers of kiwifruit, and will be helpful for the cloning of candidate genes and molecular breeding.

## 2. Results

### 2.1. Histological Characterization of Con, Fem and PT

**Figure 1:**
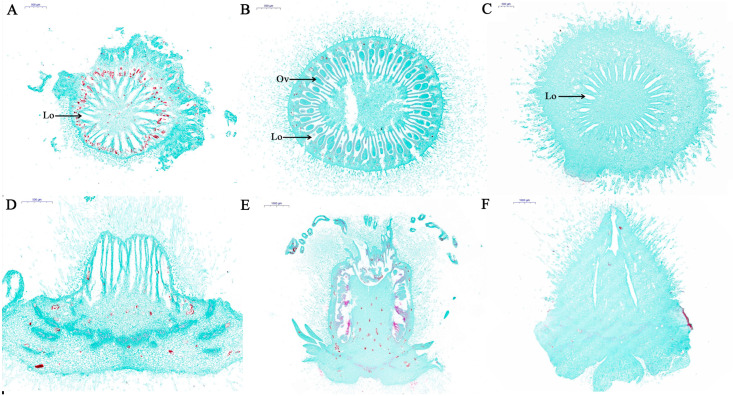
Histological characterization of Con, Fem and PT. (**A**–**C**) Transverse histological of Con, Fem and PT. (**D**–**F**) Longitudinal histological of Con, Fem and PT. Ov: Ovule, Lo: Locule. Scale bar = 500 μm (**A**–**D**); Scale bar = 1000 μm (**E**,**F**).

Normally, in non-PGR-treated kiwifruit male flowers, the pistil was necrotic and the ovary stopped developing after male flower failure. In contrast, the ovaries of PGR-treated kiwifruit male flowers were able to develop and grow (Figure S1). Therefore, we performed histological analysis of longitudinal and transverse sections of Con, Fem and PT. As shown in Figure 1, Con transverse sections displayed multiple locules within the ovary, but there was no ovule primordium produced by cell stimulation and differentiation at the suture between the carpel and the placenta within the locule, and cells located in the adaxial wall of the ovary did not divide, forming vacuolization leading to atrophy of the carpel (Figure 1A). Transverse sections of Fem revealed multiple locules within the ovary and ovules within the locules, which were anatropous and inserted on the median placenta (Figure 1B). Transverse sections of PT showed that it also does not exhibit ovule development within the locules, but its carpels developed normally without atrophy (Figure 1C). Longitudinal histological analyses showed that Fem and PT had larger sizes compared to Con (Figure 1D–F).

### 2.2. Phytohormone Level Detection

**Figure 2:**
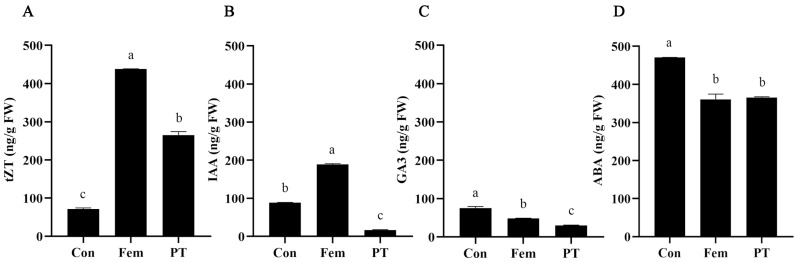
Phytohormone levels of Con, Fem and PT. (**A**) Trans-zeatin (tZT) content. (**B**) Indole-3-acetic acid (IAA) content. (**C**) Gibberellin (GA3) content. (**D**) Abscisic acid (ABA) content. Data are expressed as averages ± SD, *n* = 3. Significant differences between treatments at *p* < 0.05 are indicated by different letters over columns (Duncan’s test).

It has been previously demonstrated that ovary development requires changes in phytohormone levels and that this process is dependent on cooperation among multiple phytohormones [21]. In order to analyze the changes in phytohormone concentrations in kiwifruit ovaries after PGR treatment, the endogenous phytohormone contents in three samples were detected. As shown in Figure 2A, the contents of trans-zeatin (tZT) in PT and Fem were significantly higher than in Con. However, the contents of Indole-3-acetic acid (IAA), gibberellin (GA3) and abscisic acid (ABA) in PT were significantly lower than in Con (Figure 2B–D). These results suggest that the contents of tZT, IAA, GA3 and ABA might be closely related to ovary development.

### 2.3. RNA-Seq and Differential Gene Expression Analyses

**Figure 3:**
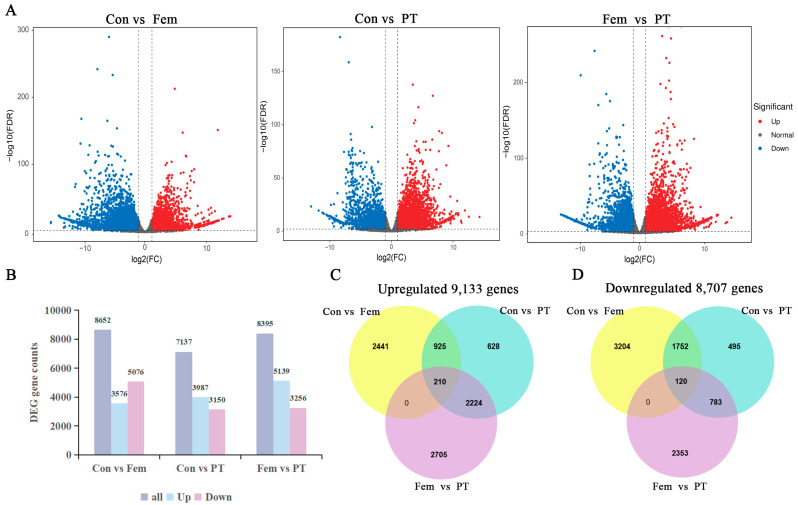
Summary of DEGs. (**A**) Volcano plot of DEGs between the three comparison groups. (**B**) Number of DEGs. (**C**) Venn diagram of the number of upregulated DEGs. (**D**) Venn diagram of the number of downregulated DEGs.

RNA sequencing of three biological replicates of Con, Fem and PT, a total of nine samples, was performed on the Illumina platform. After removing the low-quality reads, RNA-Seq generated 20.2–23.9 million clean reads for each sample (Table S1). The Q30 percentages were 92.8–93.74% and the GC contents were 46.3–46.90%, demonstrating that the quality of transcriptome sequencing data was good. To gain insight into the dynamic changes in gene expression PGR treatment, the fragments per kilobase of exon model per million mapped fragments (FPKM) of data from samples were analyzed. Differential gene expression analysis among the three kinds of samples revealed 8652 (3576 upregulated and 5076 downregulated) DEGs between Con and Fem, 7137 (3987 upregulated and 3150 downregulated) DEGs between Con and PT, and 8395 (5139 upregulated and 3256 downregulated) DEGs between Fem and PT (Figure 3A,B). Moreover, further analysis of these DEGs revealed that the upregulated DEGs included 210 core genes (Figure 3C), and the downregulated DEGs included 120 core genes (Figure 3D).

For functional annotation, the DEGs were searched using BLAST v2.14.0 against the NR, GO, KEGG, KOG, Pfam and Swiss-Prot databases. A total of 8652 DEGs between Con and Fem returning significant BLAST hits (E-value < 1.0 × 10^−5^) were annotated, including 8647 in NR, 7386 in GO, 6168 in KEGG, 4534 in KOG, 7348 in Pfam and 6670 in Swiss-Prot. A total of 7137 DEGs between Con and PT were annotated, including 7135 in NR, 6019 in GO, 5070 in KEGG, 3480 in KOG, 6146 in Pfam and 5612 in Swiss-Prot. Additionally, 8395 DEGs between Fem and PT were annotated, including 8391 in NR, 7110 in GO, 5985 in KEGG, 4222 in KOG, 7122 in Pfam and 6390 in Swiss-Prot (Figure S2).

### 2.4. GO and KEGG Enrichment Analysis of DEGs

**Figure 4:**
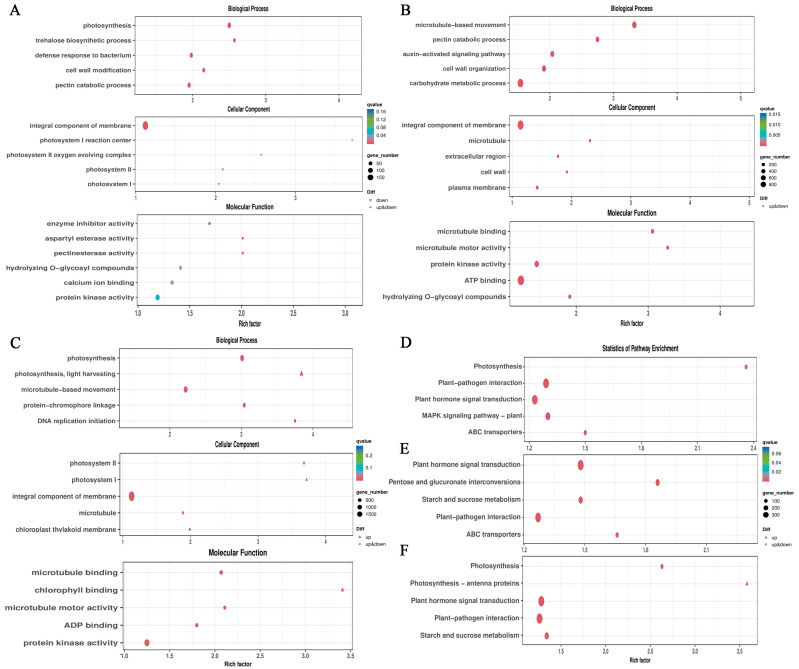
GO and KEGG enrichment analysis. (**A**) GO analysis in the Con vs. Fem combination. (**B**) GO analysis in the Con vs. PT combination. (**C**) GO analysis in the Fem vs. PT combination. (**D**) KEGG analysis in the Con vs. Fem combination. (**E**) KEGG analysis in the Con vs. PT combination. (**F**) KEGG analysis in the Fem vs. PT combination.

To investigate DEG functions, ClusterProfiler was used to analyze the GO enrichment of DEGs for biological processes (BPs), molecular functions (MFs) and cellular components (CCs), respectively, using hypergeometric tests. As shown in Figure 4A, in the Con vs. Fem combination, the most significant enriched GO categories were photosynthesis (BP), trehalose biosynthetic process (BP), defense response to bacterium (BP), enzyme inhibitor activity (MF), aspartyl esterase activity (MF), pectinesterase activity (MF), integral component of membrane (CC), photosystem I reaction center (CC) and photosystem II oxygen-evolving complex (CC). In the aspect of enrichment of DEGs for the Con vs. PT combination, the most significant enriched GO categories were microtubule-based movement (BP), pectin catabolic process (BP), auxin-activated signaling pathway (BP), microtubule binding (MF), microtubule motor activity (MF), protein kinase activity (MF), integral component of membrane (CC), microtubule (CC) and extracellular region (CC) (Figure 4B). In the Fem vs. PT comparison, the most significant enriched GO categories sequentially were photosynthesis (BP), light harvesting (BP), microtubule-based movement (BP), microtubule binding (MF), chlorophyll binding (MF), microtubule motor activity (MF), photosystem II (CC), photosystem I (CC) and integral component of membrane (CC) (Figure 4C).

To further explore the biological functions of DEGs, a KEGG enrichment analysis of DEGs was performed. For the Con vs. Fem combination, the most highly represented pathways were photosynthesis, plant–pathogen interaction and plant hormone signal transduction (Figure 4D). For the Con vs. PT combination, the most highly represented pathways were plant hormone signal transduction, pentose and glucuronate interconversions, and starch and sucrose metabolism (Figure 4E). For the Fem vs. PT combination, the most highly represented pathways were photosynthesis, photosynthesis-antenna proteins and plant hormone signal transduction (Figure 4F). Notably, plant hormone signal transduction was significantly enriched in three comparison groups, indicating that plant hormones played an important role in the regulation of ovary development.

### 2.5. Identification of Unigenes or DEGs Encoding TFs

**Figure 5:**
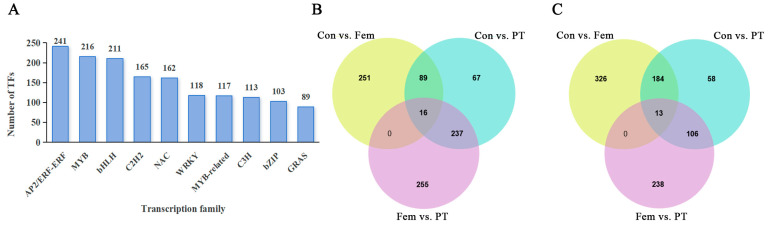
Identification of unigenes or DEGs encoding transcript factors (TFs). (**A**) Statistics of unigenes encoding TFs. (**B**) Venn diagram of upregulated DEGs annotated as TFs. (**C**) Venn diagram of downregulated DEGs annotated as TFs.

In total, 3438 unigenes were predicted to be TFs (Table S2). The top 10 TF families included AP2/ERF-ERF, MYB, bHLH, C2H2, NAC, WRKY, MYB-related, C3H, bZIP and GRAS (Figure 5A). In pairwise comparison, 880 (357 upregulated and 523 downregulated) DEGs between Con and Fem were annotated as TFs, 770 (409 upregulated and 361 downregulated) DEGs between Con and PT were annotated as TFs, and 866 (508 upregulated and 358 downregulated) DEGs between Fem and PT were annotated as TFs (Table S3). Moreover, further analysis of these DEGs revealed that the upregulated DEGs included 16 core genes annotated as TFs and these TFs were mostly in the MYB (3) and bZIP (3) families (Figure 5B, Table S3). The downregulated DEGs included 13 core genes annotated as TFs and these TFs were mostly in the AP2/ERF-ERF (2), C2H2 (2) and MADS-MIKC (2) families (Figure 5C, Table S3). These results indicate that TFs play regulatory roles in the regulation of ovary development.

### 2.6. Key DEGs Related to Phytohormone Biosynthesis and Signaling Pathways

Previous studies have shown that signaling by cytokinin and auxin is associated with ovary development [21]. KEGG analysis showed that plant hormone signal transduction pathways were significantly enriched in the three comparison groups. Therefore, we focused on DEGs related to signal transduction of cytokinin and auxin.

**Figure 6:**
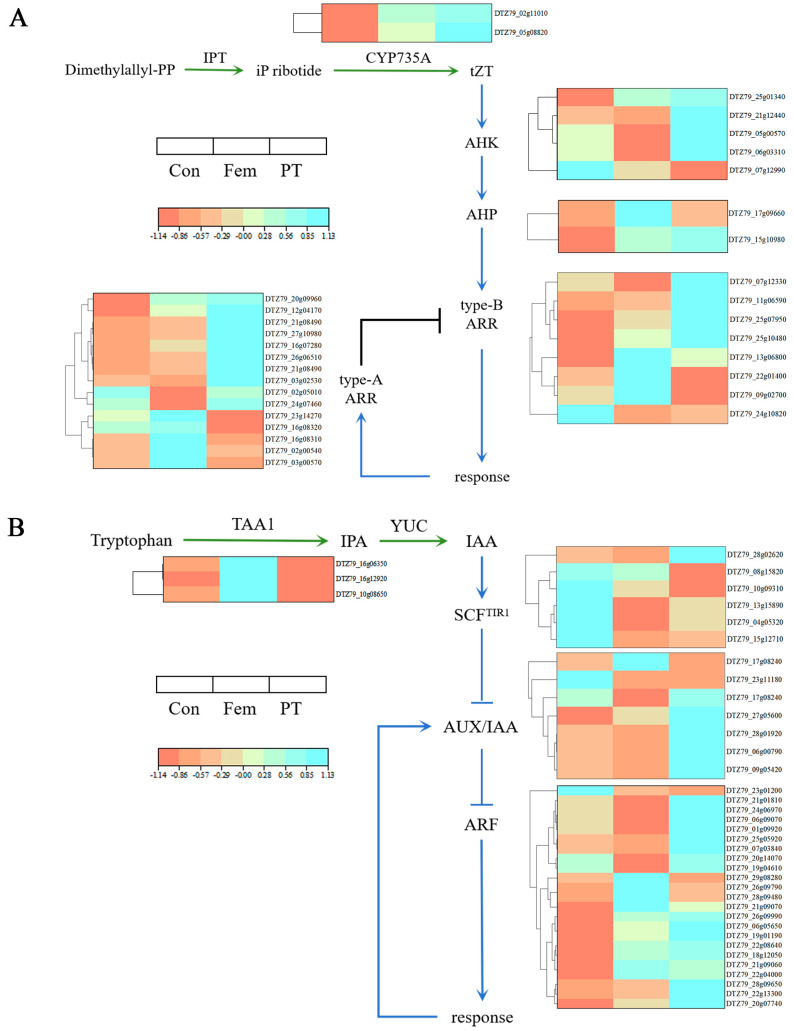
Biosynthesis and signaling pathways of plant hormones. Biosynthesis and signaling are indicated by green and blue lines. (**A**) Heatmap of DEGs involved in biosynthesis and signaling pathways related to cytokinin. (**B**) Heatmap of DEGs involved in biosynthesis and signaling pathways related to auxin.

Cytokinin is a well-known phytohormone associated with numerous aspects of plant development. There were 18 (13 upregulated and 5 downregulated), 22 (16 upregulated and 6 downregulated), and 19 (11 upregulated and 8 downregulated) DEGs in the Con vs. Fem, Con vs. PT and Fem vs. PT combinations, respectively, participating in the cytokinin-mediated signaling pathway (Table S4). The expression of *DTZ79_05g08820* and *DTZ79_02g11010*, encoding the cytochrome P450 enzymes CYP735As, assists in the conversion of isopentenyl (ip) ribotides into tZT-type cytokinin, and was significantly upregulated in PT compared to Con. Some cytokinin receptors and downstream signaling components such as *DTZ79_05g00570*, *DTZ79_25g01340*, *DTZ79_11g06590*, *DTZ79_25g10480* and *DTZ79_07g12330* were also found significantly upregulated in PT compared to Con (Figure 6A).

There were 27 (13 upregulated and 14 downregulated), 24 (18 upregulated and 6 downregulated) and 22 (15 upregulated and 7 downregulated) DEGs in the Con vs. Fem, Con vs. PT and Fem vs. PT combinations, respectively, participating in the auxin-mediated signaling pathway (Table S5). The positively regulated genes for auxin biosynthesis, *DTZ79_10g08650* and *DTZ79_16g06350*, encoding TRYPTOPHAN AMINOTRANSFERASE OF ARABIDOPSIS1 (TAA1), were significantly downregulated in PT compared to Con. In contrast, the expression of auxin receptors and auxin response factors (ARFs), *DTZ79_10g09310*, *DTZ79_15g12710*, *DTZ79_18g12050*, *DTZ79_21g01810* and *DTZ79_01g09920* was significantly upregulated in PT compared to Con (Figure 6B).

### 2.7. Analysis of DEGs Participating in Ovary Development

To investigate the mechanism of PGRs inducing ovary development in male flowers, the DEGs associated with carpel development were also investigated. The results show that there were 33 (14 upregulated and 19 downregulated), 31 (9 upregulated and 22 downregulated) and 28 (9 upregulated and 19 downregulated) DEGs in the Con vs. Fem, Con vs. PT and Fem vs. PT combinations, respectively (Table S6). For example, *DTZ79_04g03580* (SPT) and *DTZ79_19g04340* (SK41), homologs that control carpel margin meristem activity and gynoecium formation, were significantly upregulated in PT compared to Con (Table 1).

### 2.8. RNA-Seq Data Validation of Differentially Expressed Transcripts by Real-Time Quantitative PCR

**Figure 7:**
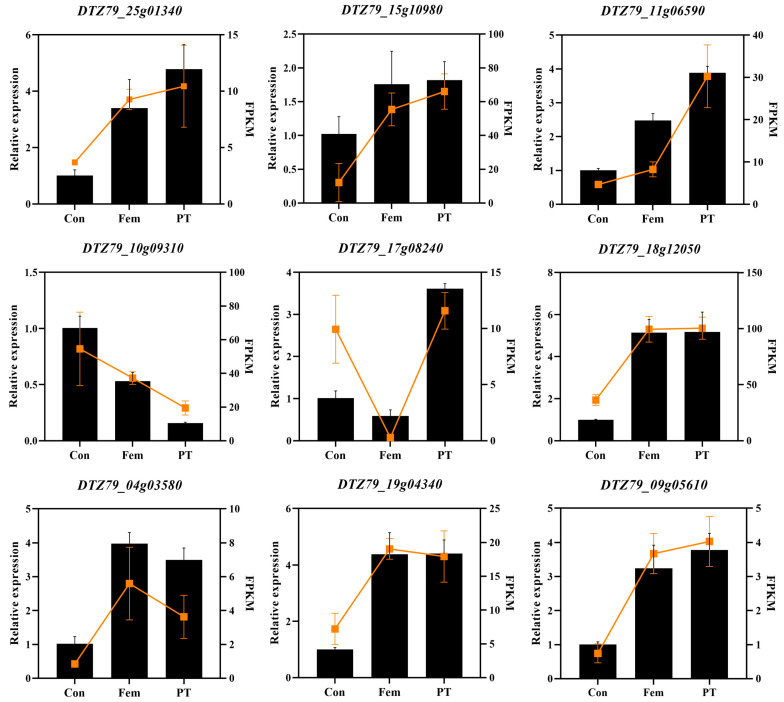
Expression patterns of DEGs based on qRT-PCR (left vertical axis) and RNA-Seq (right vertical axis). Bar graphs represent relative expression, line graphs represent FPKM values. Data are expressed as averages ± SD, *n* = 3.

To verify the accuracy of the RNA-Seq transcriptome data, three DEGs involved in cytokinin and auxin signaling, as well as three DEGs involved in carpel development, were selected and analyzed for expression by qRT-PCR, respectively. As shown in Figure 7, the expression patterns of the nine DEGs selected in the three samples were largely consistent with the corresponding expression patterns in the RNA-Seq data. This result confirms the reliability of our RNA-seq expression data and subsequent interpretation.

## 3. Discussion

Histological analyses provide insights into the causes of female and male floral sexual dimorphism in *A. eriantha* and the reasons for the rudimentary developed ovary of the male plant after externally applied PGRs. In this study, histological analysis of transverse sections of Con and Fem showed multiple locules within the ovary, but the Con locules were devoid of ovules and had atrophied carpels, whereas Fem locules had inverted types of ovules. Previous studies have displayed the characteristics of male and female flowers from early stages of development to maturity using scanning electron microscopy [3]. At the early stage of anther differentiation in female flowers, a few cells at the suture between the carpels and the placentas divided locally and circumferentially, giving rise to the ovule primordium, which further develops into ovules with the development of the flower and leads to an inverted ovule and inserted on the median placenta. In contrast, no ovule primordia were produced in the gynoecium of male flowers from bud differentiation to anthesis, and the cells in the inner wall of the whole locule showed the characteristics of dividing with enlarged nuclei, thickened cytoplasm and a neatly arranged disposition [3]. In this study, the locules of the Con were long and narrow, and the carpels were atrophied. In contrast, although there was no ovule formation in the locule of PT, its carpels developed normally without atrophy, which may be the reason why the ovary of the male plants did not atrophy from rudimentary to develop after the external application of PGRs.

Changes in endogenous phytohormone levels may have contributed to the development of ovaries in male plants. Cytokinins are promoters of cell division and differentiation, auxins regulate cell division and expansion, GA is primarily responsible for cell expansion and ABA promotes cell senescence [22,23,24,25]. PT and Fem endogenous cytokinins were higher and GA and ABA levels were lower than Con, but auxin levels of PT were lower compared to Con and Fem. The main mechanism to translate the changes in the levels of cytokinin and auxin into cellular responses was through transcriptional changes [26,27]. Therefore, in order to explore the molecular mechanisms of changes in phytohormone levels and PGR-induced ovary development, we performed transcriptional profiling to identify the genes that might contribute to PGR-induced ovary development. The results show that there were 8652 DEGs between Con and Fem, 7137 DEGs between Con and PT and 8395 DEGs between Fem and PT. KEGG pathway enrichment analysis showed that the phytohormone signaling pathway was significantly enriched in all three comparison groups, suggesting that many key genes in the phytohormone pathway were significantly altered after being treated with PGR. In this study, genes in the MYB and bZIP TF families were also greatly induced under PGR treatment. MYB proteins are key factors in regulatory networks controlling development, metabolism and responses to biotic and abiotic stresses [28]. In tomato, SlMYB21, encoding a MYB transcription factor, partially mediates the action of JA in the ovule and might control the flower-to-fruit transition [29].

The process of cytokinin biosynthesis has been relatively well studied. First, phosphoadenosine-isopentenyltransferase (IPT) transfers the isoprenyl group of dimethylallyl diphosphate to adenosine 5′-diphosphate (ADP) or adenosine 5′-triphosphate (ATP) to form ip-ribotides [30]. CYP735A then hydroxylates the ip-ribotides to a tZT-type cytokinin [31]. In this study, two CYP735As (*DTZ79_05g08820*, *DTZ79_02g11010*) were significantly more highly expressed in PT and Fem than in Con, resulting in high tZT content in PT and Fem. The cytokinin signaling pathway in plants involves multiple phosphorylation events. Histidine kinase (HK), as a cytokinin receptor, has a CHASE domain, and cytokinin binds to the CHASE domain, activating the cytoplasmic histidine kinase domain and inducing autophosphorylation of conserved His residues, resulting in the transfer of the phosphate group to the conserved Asp [32,33,34]. HK2 and HK3 are also the two main cytokinin receptors that mediate cytokinin-coordinated CMM activity during gynoecium development [35,36]. Accordingly, the expression of two HKs (*DTZ79_05g00570*, *DTZ79_25g01340*) in PT was higher than the expression in Con, and they may be involved in cytokinin-mediated ovary development. Histidine phosphotransmitters (HPs) act downstream of the HK receptor and transfer the phosphate group between the HK receptor domain and the RR receptor domain [37]. There are two types of response regulators (RRs) in the cytokinin signaling pathway: type A RRs and type B RRs. Type B RRs are able to be phosphorylated by HP to activate the Asp residue in the structural domain of the receptor, which positively regulates the transcriptional response to cytokinin [38,39]. In this study, one HP (*DTZ79_15g10980*) and three type B RRs (*DTZ79_11g06590*, *DTZ79_25g10480* and *DTZ79_07g12330*) were more highly expressed in PT compared to Con. Upregulation of these DEGs associated with cytokinin signaling may be responsible for PT development.

Tryptophan or tryptophan precursors are the beginning of auxin biosynthesis. First, TAA1 converts tryptophan to indole-3-propionic acid (IPA), and then IPA is converted to auxin by members of the YUCCA (YUC) family of flavin monooxygenases [40,41,42]. Analysis of mutant phenotypes of genes related to auxin biosynthesis suggests that normal morphogenesis of the gynoecium requires local synthesis of auxin [43]. In this study, two TAA1s (*DTZ79_10g08650*, *DTZ79_16g06350*) had a significantly lower expression in PT than Con and Fem, resulting in low IAA content in PT. Auxin-responsive gene transcription is regulated through the action of the TRANSPORT INHIBITOR RESISTANT1/AUXIN SIGNALING F-BOX (TIR1/AFB) protein, the AUXIN/INDOLE ACETIC ACID (Aux/IAA) transcriptional repressor and ARF [44]. ARF is able to bind to DNA because it has a DNA-binding structural domain, but in most cases, it does not bind to RNA directly and requires homodimerization, which is competitively inhibited by the binding of Aux/IAA deterrent proteins [45,46]. At low auxin levels, ARF binds to Aux/IAA to repress auxin-responsive genes, while at high auxin levels, auxin and TIR1/AFB protein binding induces degradation of the Aux/IAA inhibitory protein, thereby restoring ARF’s ability to homodimerize and bind to DNA [47,48,49]. ETTIN/AUXIN RESPONSE FACTOR3 (ETT/ARF3), a member of the ARF family, plays a major role in the development of gynoecium, and the reduced or absent cardiac valve organization in the *ett* mutant suggests that auxin is a key regulator of gynoecium morphogenesis [50,51]. The expression of two TIR1s (*DTZ79_10g09310*, *DTZ79_15g12710*) in PT was lower than that in Con, and the expression of two Aux/IAAs (*DTZ79_06g00790*, *DTZ79_28g01920*) and three ARFs (*DTZ79_18g12050*, *DTZ79_21g01810*, and *DTZ79_01g09920*) were higher in PT than in Con. These DEGs associated with auxin signaling are predicted to be important genes involved in ovary development in kiwifruit male plants.

DEGs associated with carpel development were also investigated. SPATULA (SPT) is a bHLH transcription factor that promotes the growth of the carpel margin and its derived tissues during the early stages of gynoecium development [52,53]. Cytokinin signaling is necessary for phloem organization properties required of CMM activation and growth, and it has recently been reported that SPT in the medial structural domain can activate such cytokinin signaling [54,55]. In addition, the expression of TAA1 and the gene encoding auxin efflux transporter protein 3 (PIN3) were regulated by SPT and cytokinins, which may produce auxin drainage important for gynoecium development [56]. SHAGGY-related protein kinases (SKs) are plant homologs of the Drosophila shaggy (SGG) gene, which is required for proper patterning of the gynoecium [57,58]. In this study, both SPT (*DTZ79_04g03580*) and SK41 (*DTZ79_19g04340*) had higher expressions in PT than in Con. These genes may play an important role in kiwifruit male plant ovary development.

In summary, the absence of atrophy in male flower ovary treated with plant hormones may be due to the normal development of the carpels induced by PGR stimulation. This process is associated with changes in endogenous phytohormone levels and phytohormone signaling. Interestingly, the differential expression of cytokinin regulation-related genes CYP735As, HP, HK and B-RR, auxin response genes TAA1, TIR1, Aux/IAA and ARF, and carpel-development-related genes SPT and SK41, suggests that these genes might play a role in the regulation of PGR-induced ovary development in male flowers. However, if we want to understand the complete regulatory network of PGR-induced ovary development in male flowers, the functions of these key candidate genes and their interactions need to be further validated in future work.

## 4. Materials and Methods

### 4.1. Plant Material and PGR Treatment

A male kiwifruit *A. eriantha* (N10-16) and a female kiwifruit *A. eriantha* (N12-16) were used for plant materials in this study. Plants were collected from the kiwifruit Germplasm Resource Nursery of National horticulture germplasm resources center. The treatment working solution was prepared with 200 mg/L CPPU solution and 1 g/L IBA solution in a certain concentration ratio. Two days before the flowers bloom, the male flowers were injected with the treatment solution. At the same time, the ovaries (receptacles and stigmatic armsremoved) were collected from the male (Con) and female (Fem) kiwifruits, and immediately frozen in liquid nitrogen. After 10 days of treatment, the ovaries (receptacles and stigmatic armsremoved) treated with PGR (PT) were collected from flowers and immediately frozen in liquid nitrogen.

### 4.2. Histological Observation

The ovaries were immediately fixed in formalin-aceto-alcohol (FAA, 10% [V/V] formaldehyde, 50% absolute ethanol, 5% acetic acid), dehydrated with a graded series of ethanol (75%, 85%, 90%, 95% and 2 × 100%), infiltrated with 100% xylene and embedded in paraffin. Serial 10 μm sections were cut with an RM2016 microtome (Leica, Shanghai, China), stained with 1% toluidine blue and visualized with a Nikon ECLIPSE E100 microscope (Nikon Instruments, Tokyo, Japan).

### 4.3. Phytohormone Analysis

Samples, ground in liquid nitrogen, were extracted using 80% aqueous methanol. Extracts were reconstituted with aqueous methanol and analyzed by liquid chromatography-tandem mass spectrometry (LC-MS/MS). Sample separation was performed on an ultra-high-performance liquid chromatography system (Waters Alliance e2695, Milford, MA, USA) with a C18 column (4.6 × 250 mm, 5 μm, 100 Å) and a 1 mL/min flow rate. We used 0.1% formic acid in water as mobile phase A and 0.1% formic acid in acetonitrile as mobile phase B. The gradient was 2–98% B in 10 min, 98–2% B in 10 min and 2% B for 2 min. An AB SCIEX 5500Q-trap mass spectrometer was used for sample detection in positive ion mode. The parameters of MS analysis were as follows: source temperature at 500 °C; ion spray volume at 5500 v; ion source gas all at 45; and curtain gas at 30. The ion pairs to be measured were detected using MRM mode. Phytohormones were extracted and their contents measured following a previously described protocol [59]. Phytohormone content was expressed in ng/g FW.

### 4.4. RNA Extraction and Illumina Sequencing

Nine ovary samples (three replicates per treatment) were directly flash-frozen in liquid nitrogen and stored at −80 °C immediately before RNA extraction. Total RNA was extracted using TRIzol^TM^ RNA extraction reagent (TIANGEN, Beijing, China) according to the manufacturer’s instructions. The integrity of RNA was accurately detected using an Agilent 2100/LabChipGX (Agilent Technologies, Santa Clara, CA, USA). Total RNA concentration in different samples was calculated using a NanoDropmicrovolume spectrophotometer (Thermo Scientific NanoDrop Products, Waltham, MA, USA). Finally, transcriptome sequencing was performed using double-end sequencing on the Illumina platform. Illumina sequencing data were deposited in the NCBI SRA database under the accession number PRJNA123406.

### 4.5. Analysis of the RNA-Seq Data

Quality reads of the raw RNA-Seq data were processed by the Fastp.0.23.4 software [Ultrafast one-pass FASTQ data preprocessing, quality control, and deduplication using fastp]. The filtered data were aligned with the kiwifruit genome sequence (https://kiwifruitgenome.org, 1 September 2019) [60]. For each gene expression, the FPKM value was calculated using StringTiev2.1.7 to estimate the gene expression level [StringTie enables improved reconstruction of a transcriptome from RNA-seq reads]. The unigene sequences obtained by RNA-Seq were aligned to the corrected transcript sequences obtained by SMRT sequencing, regarded as a reference. The DESeq Bioconductor package was used for a differential expression analysis [61]. After adjustments using the Benjamini and Hochberg approach to control for the false discovery rate (FDR), we set a *p* ≤ 0.05 and absolute value of log2 (fold-change) ≥ 1 as the thresholds for determining DEGs.

### 4.6. Functional Annotation of Transcripts and Prediction TFs

BLAST was used to annotate the unigenes by searches against the NCBI non-redundant (NR) protein database, gene ontology (GO) database [62], Kyoto Encyclopedia of Genes and Genomes (KEGG) database [63], eukaryotic ortholog groups (KOG) database, the protein family (Pfam) database [64] and Swiss-Prot database [65]. GO term with FDR ≤ 0.05 was considered significantly enriched by DEGs. Additionally, the KEGG pathway analysis was executed to retrieve the enriched pathways. iTAK: 1.7a was used to predict the plant TFs (parameters: -f 3F) [66].

### 4.7. Quantitative Real-Time PCR (qRT-PCR) Analysis

The total RNA was subsequently reverse-transcribed to quantify the desired cDNA using a ReverTra Ace qPCR RT kit (TOYOBO, Osaka, Japan) according to the manufacturer’s protocol. Some genes were selected randomly to validate the transcriptome data by RT-qPCR using LightCycler 480 SYBR Green I Master Mix. The sequences of genes were retrieved from White_genome_v1.0. The primers of the nominated genes were designed using Primer Premier 5 software. The primer sequences are listed in Supplementary Table S1. PCR amplification was performed in a 10 μL reaction volume containing 0.5 μL cDNA, 0.5 μL of each primer, 5 μL SYBR Green I Master Mix and 3.5 μL ddH_2_O. The cycling program was performed as follows: 95 °C for 5 min, followed by 40 cycles of 95 °C for 10 s, 58 °C for 15 s and 72 °C for 15s. The reactions were performed with three technical replicates and three biological replicates. The 2^−△△Ct^ method was used along with the internal reference actin gene to normalize the results.

### 4.8. Statistical Analysis

Data were analyzed using Microsoft Excel and plotted using GraphPad Prism 8.0.2 and OriginPro 2021b software. Statistical analyses were performed using SPSS 17.0 software (SPSS Inc., Chicago, IL, USA).

## Figures and Tables

**Table 1:** DEGs participating in ovary development.

Group	Gene ID	log2FC	Description
Con vs. Fem	*DTZ79_04g03580*	2.49	Transcription factor SPATULA
	*DTZ79_19g04340*	1.68	Shaggy-related protein kinase
Con vs. PT	*DTZ79_04g03580*	1.93	Transcription factor SPATULA
	*DTZ79_19g04340*	1.18	Shaggy-related protein kinase
Fem vs. PT	*DTZ79_04g03580*	−0.58	Transcription factor SPATULA
	*DTZ79_19g04340*	−0.02	Shaggy-related protein kinase

## Data Availability

Data are available from the authors upon request.

## Supplementary Materials

The following supporting information can be downloaded at https://www.mdpi.com/article/10.3390/plants14050703/s1, Figure S1: Non-PGR-treated and PGR-treated kiwifruit male flowers; Figure S2: Statistical histogram of the number of DEGs annotated in the database; Table S1: Sequencing data statistics; Table S2: Unigenes predicted to encode transcription factors (TFs); Table S3: DEGs predicted to encode transcript factors; Table S4: DEGs participating in the cytokinin biosynthesis and signaling pathway; Table S5: DEGs participating in the auxin biosynthesis and signaling pathway; Table S6: DEGs associated with carpel development; Table S7: Primers used in this study.
