# Supplementary figures and images for: RNA-Seq Analysis Reveals Potential Genes Involved in Plant Growth Regulator-Induced Ovary Development in Male Kiwifruit (Actinidia eriantha)
Source: Plants (Basel). 2025 Feb 25;14(5):703. doi: 10.3390/plants14050703 (PMC11902103; doi:10.3390/plants14050703)

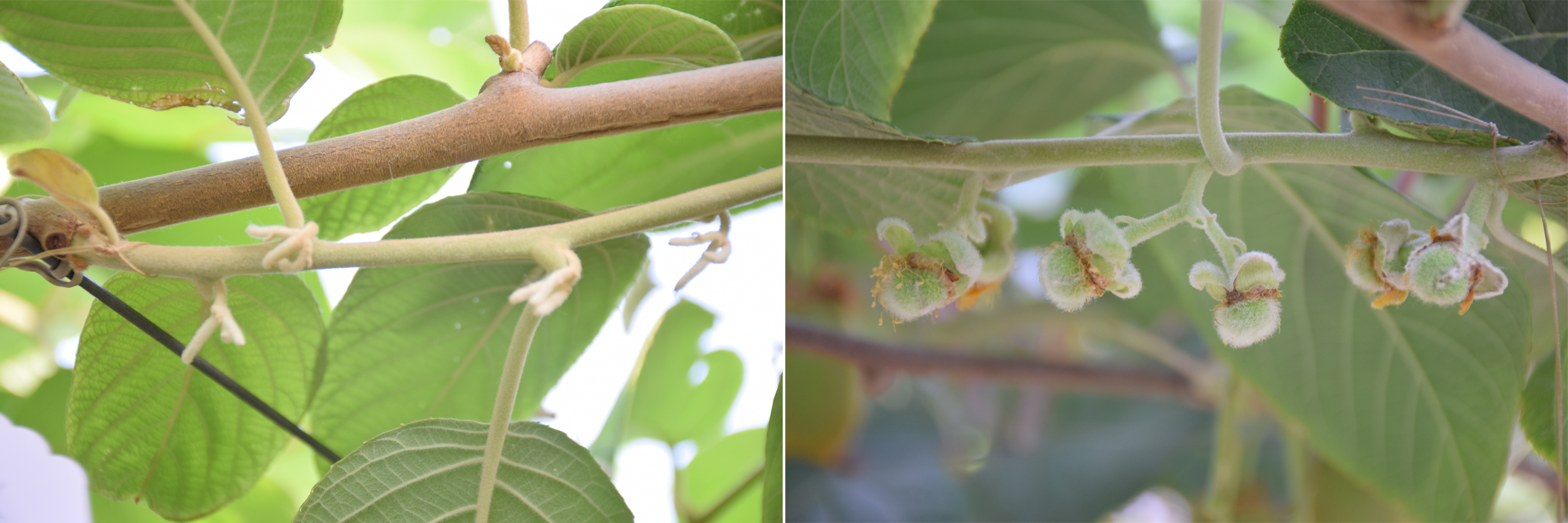

Supplement: Supplementary file 1 [file plants-14-00703-s001.zip › Figure S1.png]

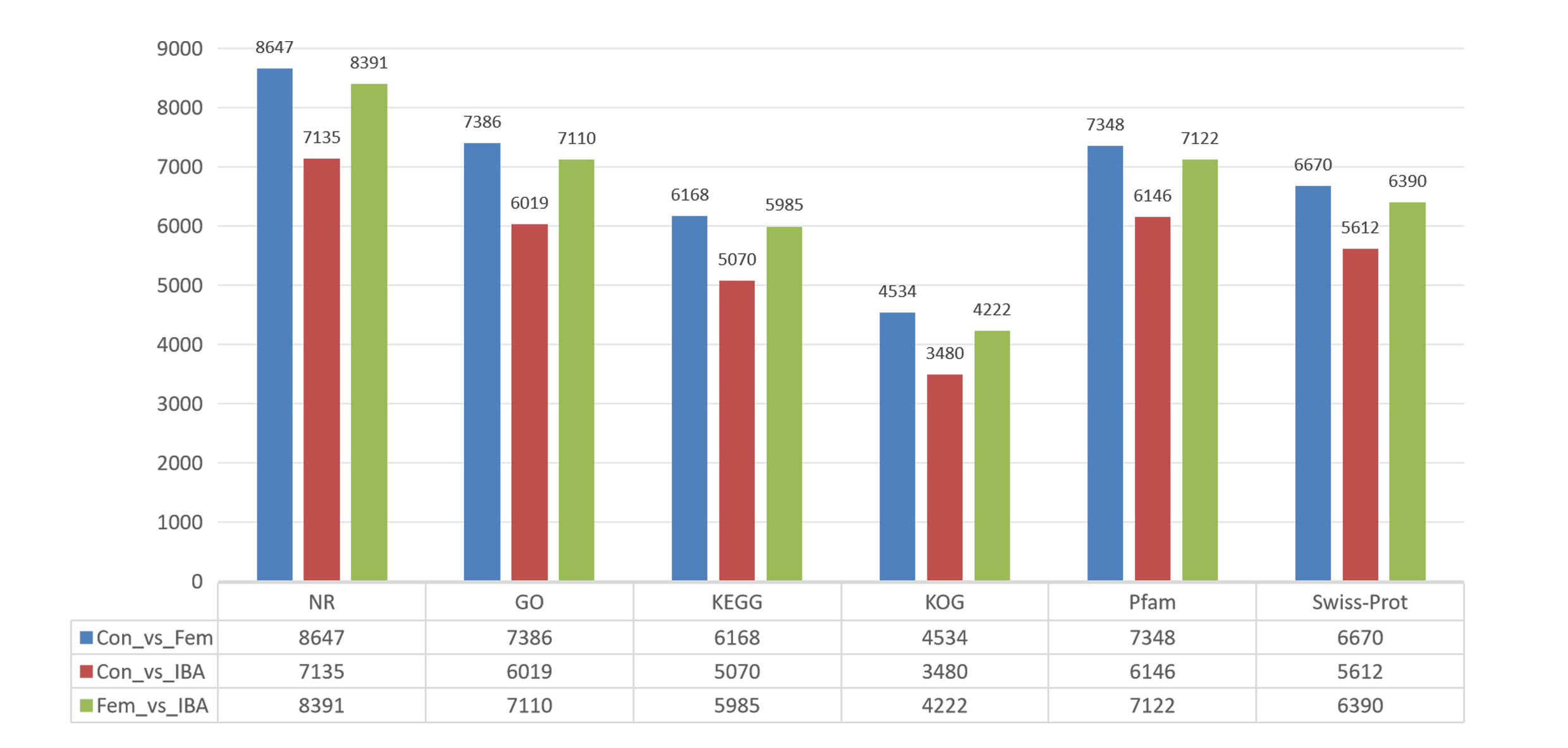

Supplement: Supplementary file 1 [file plants-14-00703-s001.zip › Figure S2.png]
